# Supplementary material for: Novel Inhibitory Role of Fenofibric Acid by Targeting Cryptic Site on the RBD of SARS-CoV-2
Source: Biomolecules. 2023 Feb 14;13(2):359. doi: 10.3390/biom13020359 (PMC9953482; doi:10.3390/biom13020359)
Supplement: Supplementary file 1 [file biomolecules-13-00359-s001.zip › biomolecules-2120248-supplementary.pdf]

Supporting Information for Novel Inhibitory Role of Fenofibric Acid by Targeting Cryptic Site on the RBD of SARS-CoV-2

Jianxiang Huang[1], Kevin C. Chan[1,2], Ruhong Zhou[1,2,3,4]*

[1]*Institute of Quantitative Biology, College of Life Sciences, Zhejiang University, Hangzhou 310027, China*

[2]*Shanghai Institute for Advanced Study, Zhejiang University, Shanghai, 201203, China*

[3]*The First Affiliated Hospital, School of Medicine, Zhejiang University, Hangzhou 310058, China*

[4]*Department of Chemistry, Colombia University, New York, NY10027, USA*

Correspondence: rz24@columbia.edu

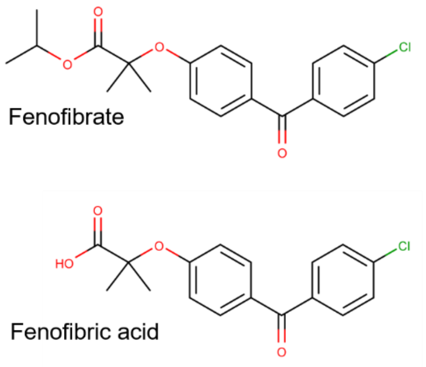

Figure S1. The chemical structures of fenofibrate and fenofibric acid.

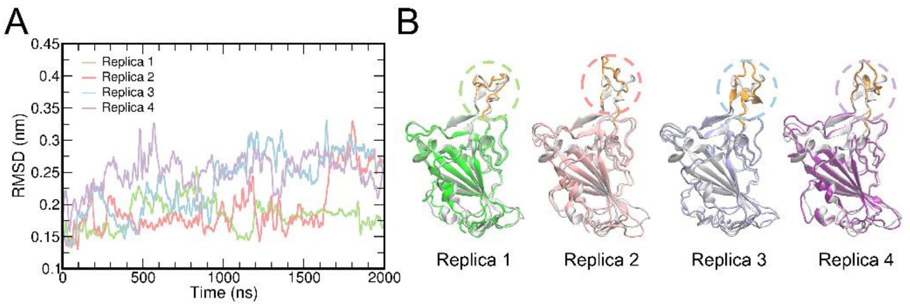

Figure S2. The RMSD results of the simulated RBD protein in the four replica systems (A). The final RBD protein structures are aligned to the initial RBD structure before the MD simulations (shown in white). The T470-F490 loops of the final RBD protein structures of replica 1 to 4 are shown in orange, while the rest of the proteins of replica 1 to 4 are displayed in green, pink, iceblue and purple, respectively (B).

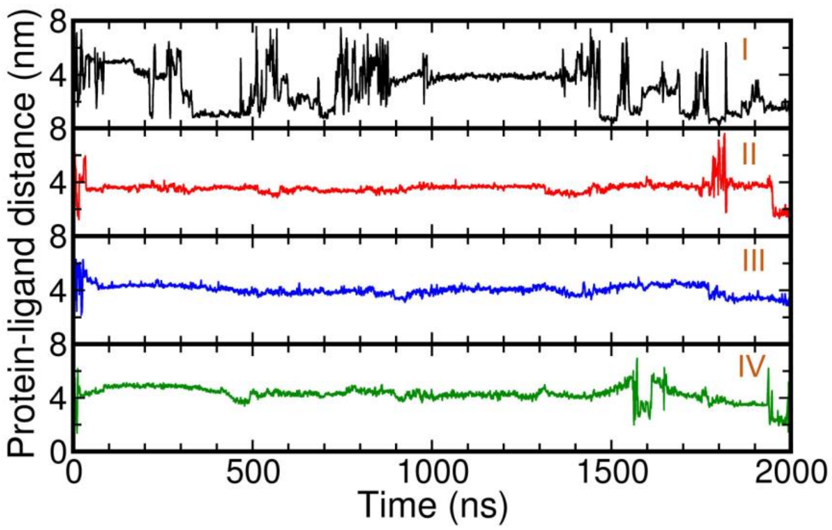

Figure S3. The COM distances between RBD and each of FAs, whereas the labels *I, II, III* and *IV* denote the data for the four FA molecules.

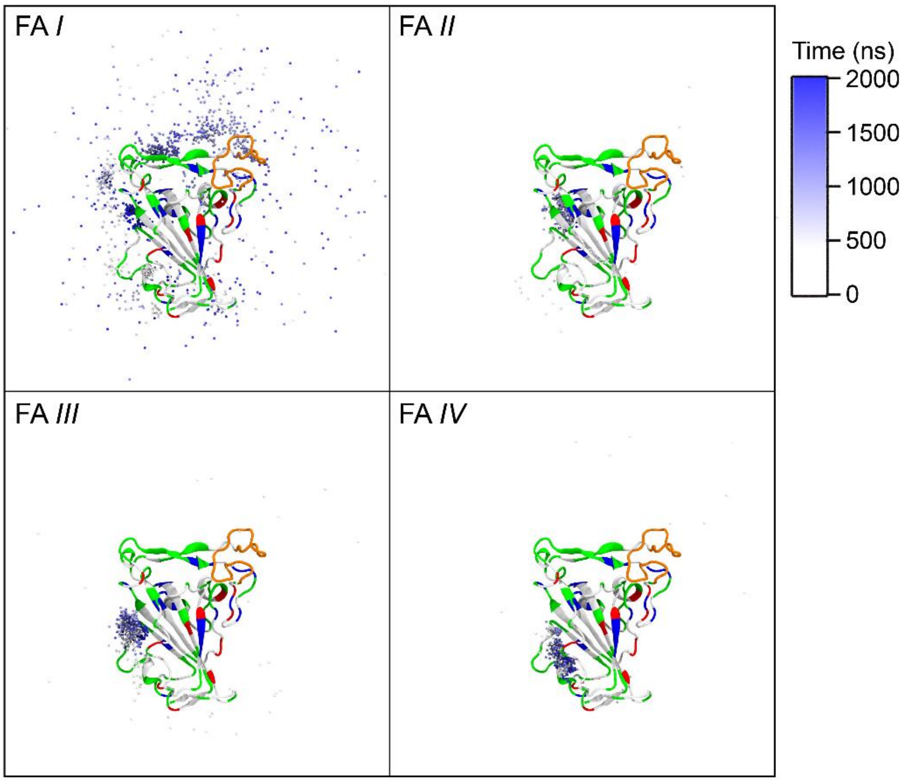

Figure S4. Time evolution of the COMs of the four FA molecules (shown as spheres). The COMs of each FA are colored according to the simulation time. COMs of frames at 0 to 2000 ns are colored from white to blue. For clarity, a total of 1250 frames with a time interval of 1.6 ns are adopted for the illustration.

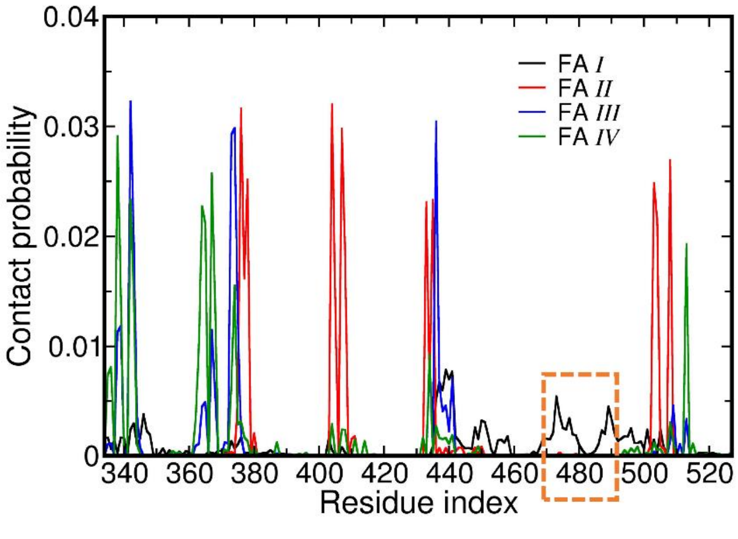

Figure S5. Contact probability of each FA molecule with the residue of the RBD protein. The calculation of contact probability is performed on the 2000 ns trajectory. To be specific, the

calculation is based on a total of 25 000 frames with a time interval of 0.08 ns. The contact between FA *I* and the T470-F490 loop of the RBD protein is marked by the dashed orange rectangle.

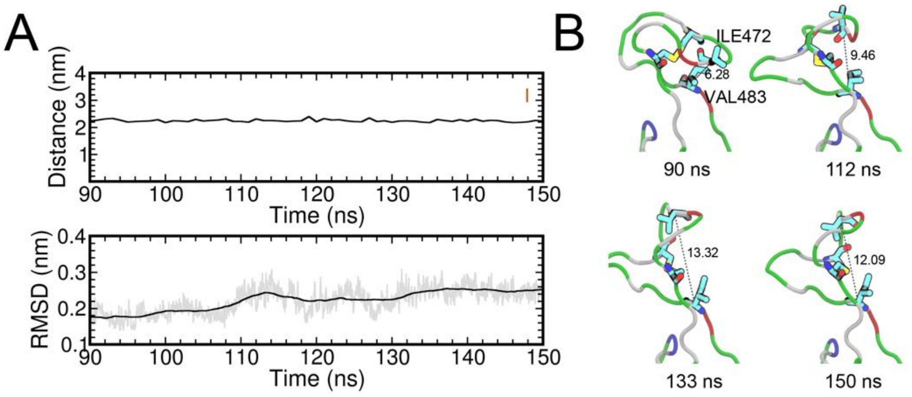

Figure S6. (A). The COM distance between RBD and FA *I* from 90 ns to 150 ns and RMSD of RBD from 90 ns to 150 ns (B). Representative snapshots of the representative fluctuating T470-F490 loop along the trajectory. Distances between alpha carbons of ILE472 and VAL483 are marked with dashed lines and the distances are labelled in angstrom. The disulfide bond near to the ILE472 and VAL483 is also displayed.

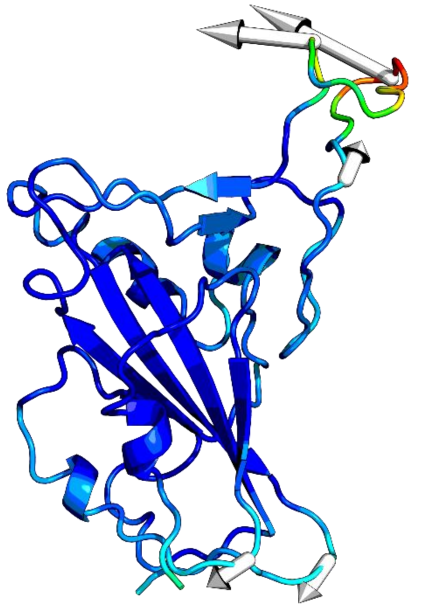

Figure S7. The ANM analysis[1] of the RBD of SARS-CoV-2 involving submission of the RBD protein structure to the ANM server (http://anm.csb.pitt.edu/). The root mean-square fluctuations of the slowest ANM modes are color mapped onto the residues. Residues with low mean-square

fluctuations are colored blue, while residues with high mean-square fluctuations are colored red. The arrow length indicates fluctuant magnitude, while the arrow orientation indicates fluctuant direction.

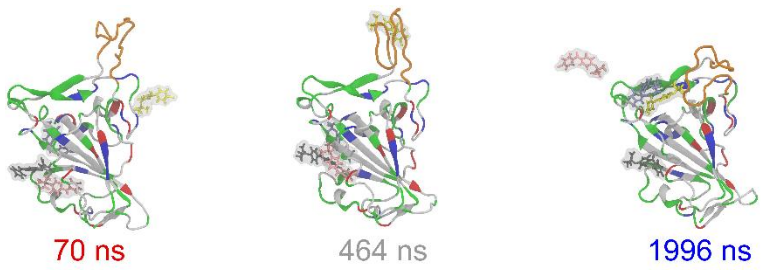

Figure S8. Representative snapshots at 70, 464 and 1996 ns for the three different trajectory projections in the PCA result.

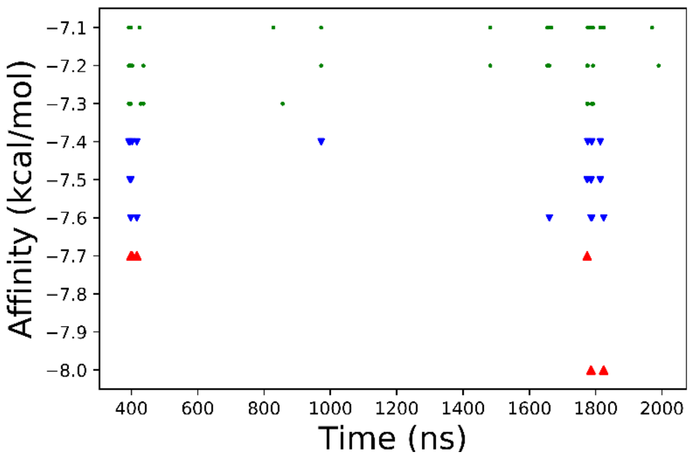

Figure S9. The FA-RBD binding affinities predicted by molecular docking calculations and data are represented by different symbols. The data with predicted binding affinities lower or equal to -7.7 kcal/mol are shown by upright red triangles. Whereas, the data with predicted binding affinities lower or equal to -7.4 kcal/mol and greater than or equal to -7.6 kcal/mol are shown as upside-down blue triangles. Similarly, the data with predicted binding affinities lower or equal to -7.1 kcal/mol and greater than or equal to -7.3 kcal/mol are shown as green dots.

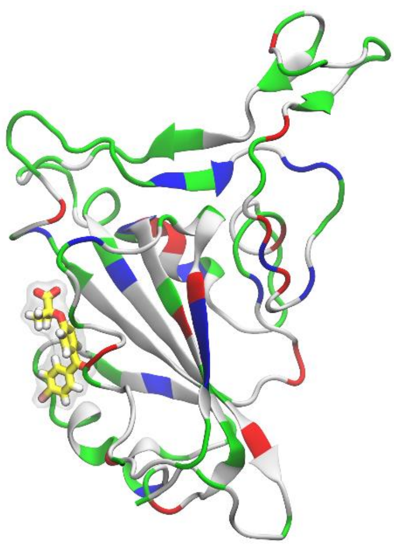

Figure S10. The selected representative docking pose on the CS1 pocket with a predicted binding affinity of -7.2 kcal/mol.

Table S1. The system details for MD simulations performed in this study

System	Number of ions	Number of water molecules	Simulation box size
RBD+4FA	58 Na+; 55 Cl-	18523	8.5×8.5×8.5 nm^3
RBD-ACE2	$1Zn^{2+}$; $238Na^+$; $215Cl^-$	74106	13.3×13.3×13.3 nm^3
FA-bound RBD-ACE2	$1Zn^{2+}$; $241Na^+$; $217Cl^-$	73993	13.3×13.3×13.3 nm^3

Table S2. The output of the CavityPlus[2] with the RBD domain of SARS-CoV-2

Cavity No.	Predicted Maximal pKd	Predicted Average pKd	DrugScore	Druggability	Vacant Volume ($Å^3$)
**1**	8.89	5.67	125	Medium	372.500
**2**	10.40	6.18	-510	Weak	321.500
**3**	7.23	5.10	-801	Weak	240.750
**4**	7.97	5.35	-1009	Weak	240.875
**5**	6.18	4.74	-1285	Weak	286.250
**6**	6.37	4.80	-1369	Weak	106.000

Pred.Avg pKd: The average value indicates the ability of a cavity binding site as a potential ligand. A value less than 6.0 suggests that this site may not be a suitable ligand-binding site. Whereas, druggability indicates the possibility of a cavity binding site to be druggable or not.[2]

Table S3. Residues of the RBD protein surrounding the cavities identified by CavityPlus[2]

Cavity No.	Residue index
**1**	LEU:335 CYS:336 PRO:337 PHE:338 GLY:339 GLU:340 VAL:341 PHE:342 ASN:343 ALA:344 THR:345 LYS:346 PHE:347 ALA:363 ASP:364 TYR:365 SER:366 VAL:367 LEU:368 TYR:369 ASN:370 SER:371 THR:372 PHE:373 PHE:374 LEU:434 TRP:436 ASN:437 THR:438 ARG:439 ASN:440 ILE:441 ASP:442 ARG:509 VAL:511
**2**	ARG:355 SER:393 ASN:394 VAL:395 TYR:396 ASP:398 LEU:425 PRO:426 ASP:427 ASP:428 PHE:429 MET:430 GLY:431 PRO:463 PHE:464 SER:514 PHE:515 GLU:516 LEU:517 LEU:518
**3**	PRO:337 GLY:339 GLU:340 VAL:341 PHE:342 ASN:343 ALA:344 THR:345 LYS:346 PHE:347 PRO:348 SER:349 ALA:352 TRP:353 GLU:354 ARG:355 LYS:356 LYS:357 ALA:397 ASP:398 SER:399 PHE:400 VAL:511
**4**	ARG:454 PHE:456 ARG:457 LYS:458 SER:459 ASP:467 ILE:468 SER:469 THR:470 GLU:471 ILE:472 TYR:473 GLN:474 ALA:475 GLY:476 CYS:480 ASN:481 GLY:482 CYS:488 TYR:489 PHE:490 PRO:491
**5**	ALA:344 THR:345 LYS:346 PHE:347 PRO:348 SER:349 VAL:350 TYR:351 ALA:352 TRP:353 GLU:354 ASN:440 ILE:441 ASP:442 ALA:443 THR:444 GLY:447 ASN:448 TYR:449 ASN:450 TYR:451 LYS:452 ARG:509
**6**	PHE:374 SER:375 THR:376 LYS:403 GLY:404 ASP:405 ASP:406 VAL:407 ARG:408 GLN:409 ILE:410 ALA:435 TRP:436 ASN:437 GLY:502 VAL:503 GLY:504 TYR:505 GLN:506 TYR:508

Table S4. The free energy results from MM/GBSA calculations of the top 17 complex structures

No.	ΔE^{ele*}	ΔE^{vdW**}	ΔG^{GB***}	ΔG^{SA****}	$-T\Delta S^{NM*****}$	ΔG (kcal/mol)
1	-85.67	-40.64	92.48	-4.87	18.31	-20.38
2	-108.80	-24.63	104.83	-3.73	12.93	-19.39
3	-69.87	-36.61	77.01	-4.64	15.62	-18.50
4	-32.33	-38.70	34.31	-4.93	24.20	-17.46
5	-101.85	-32.80	104.18	-4.44	19.25	-15.65
6	-77.62	-29.78	83.95	-3.97	13.24	-14.18
7	-88.72	-28.33	94.41	-3.83	12.87	-13.61
8	-60.70	-30.49	59.70	-3.78	21.99	-13.28
9	-93.71	-28.82	99.97	-3.82	14.10	-12.28
10	-118.98	-28.06	124.75	-3.86	14.42	-11.73
11	-49.99	-25.00	53.85	-3.56	14.27	-10.43
12	-94.45	-34.66	104.71	-4.79	18.92	-10.29
13	-74.69	-32.18	83.02	-4.36	18.42	-9.79
14	-121.65	-31.46	132.65	-4.29	16.18	-8.57
15	-126.93	-28.90	132.21	-4.14	20.98	-6.78
16	-104.00	-26.43	109.74	-3.92	19.33	-5.29
17	-74.94	-23.80	83.57	-3.19	16.18	-2.17

*: ΔE^{ele}: coulombic energy.

**: ΔE^{vdW}: van der Waals energy.

***: ΔG^{GB}: generalized Born polar solvation energy.

****: ΔG^{SA}: non-polar solvation energy.

*****: $-T\Delta S^{NM}$: entropy term calculated using normal mode analysis.

Table S5. The free energy results from MM/GBSA calculations based on the MD simulations of the representative pose on CS1

ΔE^{ele}	ΔE^{vdW}	ΔG^{GB}	ΔG^{SA}	$-T\Delta S^{NM}$	ΔG (kcal/mol)
-88.25	-24.03	92.55	-2.93	20.26	-2.40

References

[1] E. Eyal, G. Lum, I. Bahar, The anisotropic network model web server at 2015 (ANM 2.0), Bioinformatics 31(9) (2015) 1487-1489.
[2] Y. Xu, S. Wang, Q. Hu, S. Gao, X. Ma, W. Zhang, Y. Shen, F. Chen, L. Lai, J. Pei, CavityPlus: a web server for protein cavity detection with pharmacophore modelling, allosteric site identification and covalent ligand binding ability prediction, Nucleic Acids Res. 46(W1) (2018) W374-W379.
