# Supplementary figures and images for: Novel Inhibitory Role of Fenofibric Acid by Targeting Cryptic Site on the RBD of SARS-CoV-2
Source: Biomolecules. 2023 Feb 14;13(2):359. doi: 10.3390/biom13020359 (PMC9953482; doi:10.3390/biom13020359)

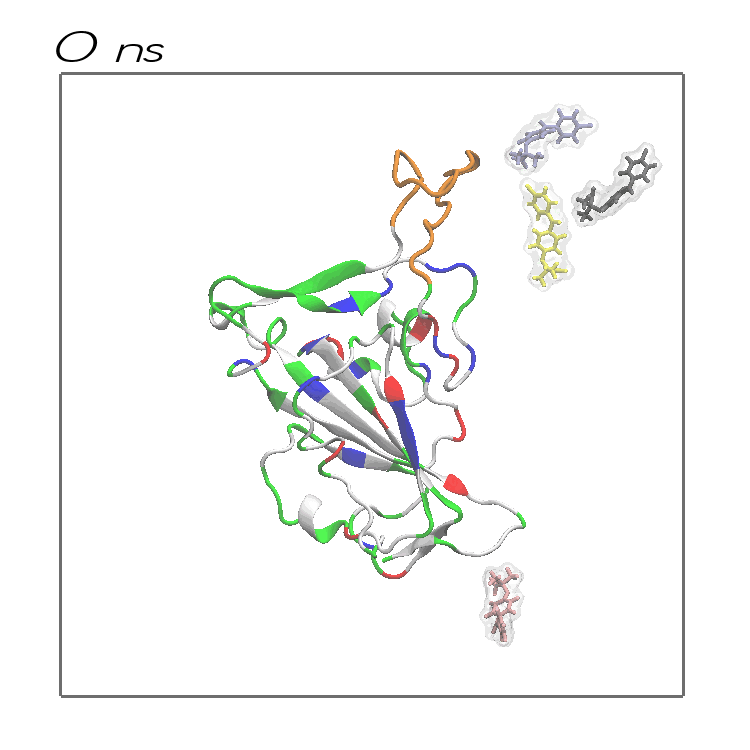

Supplement: Supplementary file 1 [file biomolecules-13-00359-s001.zip › Movie S1.gif]
